# Dissociation in pediatric population: risk factors and phenomenology. A scoping review

**DOI:** 10.1192/j.eurpsy.2026.11118

**Published:** 2026-07-31

**Authors:** N. Szejko, A. Dunalska, A. Jakubczyk, A. Lewandowska

**Affiliations:** 1 Medical University of Warsaw, Warsaw; 2 Babinski Hospital, Lodz, Poland

## Abstract

**Introduction:**

Dissociation is a transdiagnostic symptom characterized by sensation of disconnect to own body or surroundings. It can range from mild daydreaming to severe detachment from reality and often develops as a psychological response to overwhelming stress or trauma. While it can occur across the lifespan, it is an underinvestigated topic in pediatric population.

**Objectives:**

The purpose of this scoping review is to present an overview of the prevalence, phenomenology and psychopathology related to the phenomenon of dissociation in children.

**Methods:**

We have organized our findings, where appropriate, according to age range (pre-school, early school age, middle childhood, early adolescence and middle to late adolescence). Scoping review has been conducted in accordance with JBI methodology and abstracts have been screened by two independent reviewers.

**Results:**

Our search indicated that, similarly to adults, children experience variety of dissociative symptoms such as depersonalization, derealization and dissociative amnesia, peritraumatic and physiological dissociation, while other types of dissociative disorders including identity confusion are very rare and controversial in children given an evolving nature of identity in this group. Main risk factors for development of dissociation in children include early trauma (physical, sexual, or emotional abuse), neglect, witnessing domestic violence, attachment disruptions (separation from caregiver) and chronic stress especially in relation to traumatic event (war, displacement). There are several dissociation-specific scales that could be used in pediatric population, such as Child Dissociative Checklist, Adolescent Dissociative Experiences Scale, Child Dissociation Interview, Structured Clinical Interview for DSM-IV Dissociative Disorders – Child Version, Trauma Symptom Checklist for Children and Multidimensional Inventory of Dissociation – Child/Adolescent Version. From the clinical perspective, in younger children dissociation can manifest as spacing out or “freezing”, unusual play (e.g., traumatic reenactments), but also sudden regression, while in older children feeling unreal, “numb”, losing time or forgetting actions is more frequent. Main diagnoses associated with dissociation in children are post-traumatic stress disorder (PTSD), functional neurological disorder and borderline personality development. Main therapeutic approaches in children include: trauma-focused cognitive-behavioral therapy, play therapy, family therapy and, in rare cases, Eye Movement Desensitization and Reprocessing. In case of comorbidities, tailored pharmacotherapy also plays a role.

**Conclusions:**

Our findings underscore that dissociation in children, while frequently underrecognized, constitutes a clinically meaningful and trauma-associated phenomenon that necessitates developmentally sensitive diagnostic approaches and early, trauma-informed therapeutic strategies.

**Disclosure of Interest:**

None Declared

